# Microbiota of semen from stallions in Sweden identified by MALDI-TOF

**DOI:** 10.1016/j.vas.2020.100143

**Published:** 2020-10-01

**Authors:** Ziyad Al-Kass, Erik Eriksson, Elisabeth Bagge, Margareta Wallgren, Jane M. Morrell

**Affiliations:** a Department of Clinical Sciences, Swedish University of Agricultural Sciences, Box 7054, SE-75007 Uppsala, Sweden; b Department of Bacteriology, National Veterinary Institute SE-751 89, Uppsala, Sweden; c Department of Surgery and Theriogenology, College of Veterinary Medicine, university of Mosul, Mosul, Iraq

**Keywords:** Equine semen, Microorganism, Non-pathogenic bacteria, MALDI-TOF, Semen extender, Contaminants

## Abstract

Stallion semen is known to contain environmental bacteria and normal commensals, and in some cases may contain opportunistic pathogens. These bacteria may negatively influence sperm quality during storage before artificial insemination. The bacteria isolated depend on the culture conditions and method of identification; therefore, the aim of this study was to identify as many of the bacteria present in stallion semen as possible by culturing aliquots of semen under a variety of conditions. Eleven semen samples were available: five extended semen samples from one stud together with a sample of the extender, and six raw semen samples from another stud. Aliquots of semen samples were cultured on different agars and under specialized conditions; individual bacterial colonies were identified using Matrix-assisted laser desorption ionization time of flight mass spectrometry. Approximately 55% of the bacteria could be identified, with 20 bacterial taxa being isolated from semen samples from the five stallions on the first stud and 11 taxa from the semen samples from six stallions on the second stud. *Staphylococcus* spp. were present in all samples, and *Micrococcus* spp. were present in all of the extended semen samples although they were also isolated from the extender. The number of bacteria in colony forming units per mL varied considerably among samples. Only one microbe known to be associated with equine infertility, *Pseudomonas* spp., was isolated from three samples, albeit in low numbers. In conclusion, bacterial culture followed by MALDI-TOF does not identify all bacteria present in stallion semen samples. In-depth knowledge of which microbes are likely to be present is useful in determining their effects on sperm quality and, where appropriate, developing protocols for effectively controlling microbial growth.

## Highlights

• Bacteria isolated from stallion semen vary in number and species found.
• Differences may depend on culture conditions and method of identification.
• 64% bacteria in stallion semen were cultured and identified by MALDI-TOF.
• The number of bacteria varied considerably among samples, even from the same stud.
• More information is needed on their effect on sperm quality.

## Introduction

Bacteria are normally found on the genital organs and skin of the abdomen in stallions, where they form part of the normal flora. Semen becomes contaminated with bacteria as it passes out of the male reproductive tract. The reproductive tract of the mare has a well-defined response to deal with bacteria deposited during mating (Christoffersen & Troedsson, 2107) which functions well unless the bacterial load is very high. Some bacteria, however, are capable of causing disease in mares, such as *Taylorella equigenitalis* (the causative agent of Contagious Equine Metritis [CEM]), *Pseudomonas aeruginosa*, and *Klebsiella pneumoniae* (Blanchard, Kenny & Timony, 1992). In addition, *Escherichia coli, Streptococcus equisimilis, Streptococcus zooepidemicus, Bacillus* spp. (Parlevliet & Samper, 2000; Samper & Tibary, 2006), *Mycoplasma equigenitalium, M. subdolum* and *Acholeplasma* spp. are responsible for cases of infertility, endometritis and abortions in mares (Heitmann, Kirchhoff, Petzoldt & Sonnenschein, 1979; Kirchhoff, Heitmann & Bisping, 1980).

Bacteria are also a cause for concern in semen for artificial insemination. The semen extender used to maintain sperm viability also serves as a nutrient broth to support bacterial multiplication, and these bacteria are transferred to the mare during insemination. They may also affect sperm quality, particularly during storage. Therefore, antibiotics are routinely added to commercial semen doses to prevent multiplication of the contaminating bacteria (Morrell & Wallgren, 2014). This use of antibiotics is problematic since it is non-therapeutic and may contribute to antibiotic resistance in bacteria, which is a global problem. Therefore, knowledge of the bacteria that are present in semen and what effect they have on sperm quality and on the inseminated mare, is needed to optimize how they can be controlled (Althouse, Skaife & Loomis, 2010; Aurich & Spergser, 2007).

Different methods are used to isolate and identify aerobic and anaerobic bacteria. The standard method to identify bacteria includes culturing under specific conditions (Harrigan, 1998), using selective agar to obtain more accurate results (Speck, Ray & Read, 1975). However, some bacteria are slow growing, some require special media or specific culture conditions, and over-growth of fast-growing bacteria may prevent others from growing (Patel, 2001). In addition, culturing bacteria is time-consuming, requiring at least 24 to 48 h for most aerobic bacteria and sometimes much longer for fastidious or slow-growing ones.

Once bacteria have been cultured, they require identification.. Traditionally, colony morphology and Gram-stain have been used to identify bacteria (Carter, 1990) together with biochemical tests; several studies on bacteria in stallion semen have used these techniques for identification (Neto et al., 2015; Ortega‐Ferrusola et al., 2009; Varner et al., 1998). Recently, faster and more accurate methods to identify bacterial species have become available, such as Matrix-assisted laser desorption ionization time of flight mass spectrometry (MALDI-TOF MS) (Biswas & Rolain, 2013). This method, which is based on the protein structure of the isolate, is now routinely used in many clinical microbiology laboratories (Bizzini & Greub, 2010; Croxatto, Prod'hom & Greub, 2012; Seng et al., 2010) since it is fast, simple, reliable and cheap (apart from the purchase of the instrument itself). It is particularly useful for environmental bacteria. Therefore, it might be possible to identify the bacterial flora of stallion semen more precisely using this technology.

The aims of this study were as follows: 1) To identify commensal and potentially pathogenic bacteria in semen from stallions in Sweden by means of conventional culture and MALDI-TOF MS; and 2) to investigate the individual variation in bacterial taxa in semen from stallions on the same stud.

## Material and methods

### Study design

The stallions were housed in individual boxes bedded on straw (Group A) or shavings (Group B), with daily exercise and access to a paddock. All stallions were fed concentrates and minerals and had access to water ad libitum from a drinking bowl; stallions in Group A were also provided with silage whereas those in Group B received hay. These two studs were chosen because of their location (relatively short travelling time to the laboratory at SVA) and the presence of several stallions on the premises, making possible a comparison among individuals on the same stud.

Semen samples were collected from five adult stallion (7 to 17 years) from a commercial stud in Sweden (Group A). After the animals had mounted a phantom, semen was collected using a Missouri artificial vagina fitted with an inline filter to remove gel. Each stallion had its own AV, which was washed and sterilized with alcohol after each collection, and stored in a cupboard. The collecting flask was washed, dried, covered with foil and stored before placing in the incubator prior to semen collection. A disposable liner and filter were used, and the gloves worn by the semen collector were disposable.

Immediately after collection, the semen was extended with EquiPlus (Minitüb, Tiefenbach, Germany) to a final sperm concentration of 100 × 10^6^ /mL. This extender does not contain antibiotics. Sperm concentration was measured at the time of collection using a Nucleocounter-SP 100 (Chemometec, Allerød, Denmark). The semen was transferred to sterile tubes and placed on ice for transfer to the Swedish National Veterinary Institute (SVA), together with an aliquot of the extender. Transport time was approximately two hours.

In addition, raw semen samples were available from six stallions aged 5–17 years at another stud (Group B). The semen samples were collected with a Missouri artificial vagina, excluding the gel fraction. They were immediately transported on ice in sterile tubes to SVA; the transport time was approximately 60 min.

### Bacteriology

Each sample (semen samples plus an aliquot of extender alone for Group A samples) was serially diluted, plated on different agars, and cultured under aerobic and anaerobic conditions, as follows: Fastidious anaerobe agar (FAA) plates were incubated at 37 °C for 2 days in anaerobic conditions in a bell jar with gas packs (AnaeroGen® Oxoid, Basingstoke, Hampshire, England), Blood agar, MacConkey agar, Mannitol salt agar and Pseudomonas isolation agar (PIA) were incubated at 37 °C for 2 days under aerobic conditions. Chocolate agar and colistine oxolinic blood agar (COBA, streptococcal selective agar) were incubated at 37 °C for 2 days in microaerophilic conditions in a separate incubator. Blood agar plates contained defibrinated horse blood. For calculating bacteria concentration, nutrient agar plate count agar (PCA) was incubated at 30 °C for 3 days, in microaerophilic conditions. The microaerophilic conditions were created by supplying CO_2_ to the incubator from a gas cylinder; an alarm was set to alert the operator to low CO_2_ levels and the time when the door was opened was kept to a minimum. All agar plates were provided by the laboratory at SVA.

### Bacterial identification

Agar plates were checked after incubation and bacterial colonies identified according to their morphology i.e. shape and appearance; three to five colonies of similar appearance were chosen for sub-culture. The number of colonies was calculated to determine the bacterial load, by counting the number of bacteria in serial dilutions. The bacteria were sub-cultured, and incubated at 37 °C for 24 h under the appropriate conditions.

After sub-culture, single bacterial colonies were picked for identification using the Bruker Biotyper MALDI-TOF system. Sub-cultured colonies were applied on clean 96 target plates, in duplicate. Samples were overlaid with a matrix solution and air-dried. The target plates were run on the MALDI-TOF instrument in order to acquire profile spectra which were compared to spectra in the Bruker database for identification, using the latest version of the database. .

The bacteria were identified to species level if MALDI-TOF analyses presented a score ≥ 2.0 and to genus level with a score ≥1.7.

## Results

### Group A – extended semen samples from stud 1

It was possible to identify 55 isolates to species level and 66 to genus level (64%). The remaining 70 isolates were not present in the MALDI-TOF database.

Bacteria present in all semen samples from this stud were *Micrococcus* spp and *Staphylococcus* spp., (although these bacteria were also found in the extender), as well as *Mycoplasma* spp., *Corynebacterium* spp., and *Bacillus* spp. A number of other taxa were isolated, median 9 per extended semen sample (range 7 – 11). The number of microbes isolated on different agar plates is shown in Table 1 and summarized in Table 2. The highest bacterial count on PCA was 189,000 colony forming units/ml (cfu/mL) in stallion 3, whereas the lowest was 40,700 cfu/mL in stallion 5. Extended semen samples from stallions 1, 2 and 4 had 915,000, 102,000, and 100,500 cfu/mL respectively; the semen extender contained 80 cfu/mL (Table 1).

**Table 1:** Number of bacteria (colony-forming units/mL) from extended stallion semen isolated on different agar plates (Group A).

Agar plate	Bacteria	Stallion 1	Stallion 2	Stallion 3	Stallion 4	Stallion 5	extender
**Blood**	*Aerococcus* spp.	1000	200	0	10	800	0
	*Staphylococcus* spp.	9000	100	0	250	35	1
	*Bacillus* spp.	0	0	0	0	10	1
	*Acinetobacter* spp.	5000	0	0	0	0	0
	*Streptococcus* spp.	10,000	0	2500	0	0	0
	*Psychobacter* spp.	10,000	0	0	0	0	0
	*Advenella* spp.	12,500	0	0	0	0	0
	*Neisseria* sp.	100	0	0	0	0	0
	*Oligella* spp.	0	200	2000	0	0	0
	*Micrococcus* spp.	0	20	0	0	0	20
	*Brevibacterium* spp.	0	0	21,000	100	0	0
	*Arthrobacter* spp.	0	0	100	0	0	0
	*Corynebacterium* spp.	0	0	0	200	0	2
**Chocolate**	*Micrococcus* spp.	1000	0	30,000	200	1100	7
	*Aerococcus* spp.	2000	100	0	0	400	0
	*Staphylococcus* spp.	0	0	3000	550	100	10
	*Acinetobacter* spp.	3000	0	40,000	0	0	0
	*Kocuria* sp.	200	0	0	0	0	0
	*Mycoplasma* spp.	0	0	20,000	0	0	10
	*Oligella* spp.	0	0	0	4000	0	0
	*Kytococcus* sp.	0	0	0	100	0	0
**COBA**	*Staphylococcus* spp.	0	0	0	0	150	0
	*Aerococcus* spp.	0	10	0	0	0	0
**FAA**	*Corynebacterium* spp.	0	7000	0	0	0	0
	*Propionobacterium* spp.	0	1600	0	3000	0	0
	*Bacteroides* spp.	0	0	0	100	0	0
	*Streptococcus* spp.	0	0	200	0	0	0
	*Acinetobacter* spp.	2000	0	10	1	16	0
**MacConkey**	*Advenella* spp.	2000	0	0	0	10	0
	*Pseudomonas* spp.	0	0	0	0	1	0
	*Oligella* spp.	0	0	100	0	0	0
**MAST**	*Staphylococcus* spp.	5000	0	0	900	0	1
**PIA**	*Pseudomonas* spp.	1	2	0	0	1	0
	*Serratia* sp.	4	0	0	0	0	0
**PCA**		915,000	102,000	189,000	100,500	40,700	80

Note: COBA = colistine oxolinic blood agar;.

FAA = Fastidious anaerobe agar.

MAST = mannitol salt agar with lithium chloride (selective for staphylococci).

PIA = Pseudomonas isolation agar.

PCA = plate count agar.

**Table 2:** Summary of total number (colony-forming units/mL) of each bacterial taxa isolated from extended stallion semen and the number isolated on Plate Count Agar.

Bacteria	Stallion 1	Stallion 2	Stallion 3	Stallion 4	Stallion 5	extender
*Acinetobacter* spp.	10,000	0	40,010	1	16	0
*Advenella* spp.	14,500	0	0	0	10	0
*Aerococcus* spp.	3000	310	0	10	1200	0
*Arthrobacter* spp.	0	0	100	0	0	0
*Bacillus* spp.	0	0	0	0	10	1
*Bacteroides* spp.	0	0	0	100	0	0
*Brevibacterium* spp.	0	0	21,000	100	0	0
*Corynebacterium* spp.	0	7000	0	200	1100	2
*Kocuria* sp.	200	0	0	0	0	0
*Kytococcus* sp.	0	0	0	100	0	0
*Micrococcus* spp.	1000	20	30,000	200	1100	27
*Mycoplasma* spp.	0	0	20,000	0	0	10
*Neisseria* sp.	100	0	0	0	0	0
*Oligella* spp.	0	200	2100	4000	0	0
*Propionobacterium* spp.	0	1600	0	3000	0	0
*Pseudomonas* spp.	1	2	0	0	2	0
*Psychobacter* spp.	10,000	0	0	0	0	0
*Serratia* sp.	4	0	0	0	0	0
*Staphylococcus* spp.	14,000	100	3000	1700	285	12
*Streptococcus* spp.	10,000	0	2700	0	0	0
**Grand total identified by MALDI-TOF**	62,805	9232	118,910	9411	2623	52
**Total on Plate Count Agar**	915,000	102,000	189,000	100,500	40,700	80

### Group B- raw semen samples from stud 2

In total, 11 taxa were isolated and identified from raw semen samples (Table 3)with a median 0f 3.5 taxa per sample (range 2 to 6). Approximately 45% of the isolates could be identified by MALDI-TOF. Again, Staphylococci were identified in all of the samples, although the other bacteria differed among stallions. Four genera that had not been found in the samples from stallions in Group A were identified among the samples from stallions in Group B: *Fusobacterium, Paenibacillus, Brevibacillus* and *Pantoea*.

**Table 3:** Number of bacteria (colony-forming units/mL) from raw stallion semen isolated on agar plates (Group B).

stallion	1	2	3	4	5	6
*Staphylococcus* spp.	10,000	8000	4000	6000	8000	12,000
Bacillus spp.	100	0	150	100	0	50
*Corynebacterium* spp.	1000	0	1500	0	0	0
*Micrococcus* spp.	50	0	0	200	100	0
*Fusobacterium* spp.	0	400	0	0	0	0
*Mycoplasma* spp.	0	0	0	0	0	5000
*Pantoea* spp.	0	0	0	0	50	0
*Brevibacillus* spp.	0	0	0	0	3000	0
*Paenibacillus* spp.	0	0	0	1000	0	0
*Acinetobacter* spp.	0	0	0	800	0	0
*Aerococcus* spp.	0	0	0	500	0	0

Note: approximately 45% of the bacteria isolated could be idenitifed by MALDI-TOF.

## Discussion

In this study, total bacterial counts varied between stallions, in agreement with most previous studies (Al-Kass, Eriksson, Bagge, Wallgren & Morrell, 2019; Ortega‐Ferrusola et al., 2009). *Staphylococcus* spp. were isolated from all stallion semen samples, in agreement with most previous studies on fresh semen (Al-Kass et al., 2019; Corona, Cossu, Bertulu & Cherchi, 2006; Ortega‐Ferrusola et al., 2009; Pasing et al., 2013) and even in frozen semen (Corona & Cherchi, 2009; Neto et al., 2015). However, in contrast to our previous study on semen from pony stallions in Austria (Al‐Kass et al., 2018), *Corynebacterium* spp. were detected in only three of the 11 samples in the present study. These differences could be due to individual variation among stallions or to differences in husbandry conditions and handling regimens.

Apart from Staphylococci, the bacterial taxa identified in semen from stallions in group A and the number of bacteria present varied considerably among stallions. Half of the taxa isolated from the extended semen samples were environmental in origin, being commonly found in soil and water (vetbact website, 2020). The remaining taxa were *Staphylococcus* and *Streptococcus*, which are commensals on skin, mucus membranes and the respiratory tract; *Kytococcus* and *Propionobacterium*, which are commensals on skin Al‐Kass et al. (2018); *Neisseria* which is a commensal of mucosa (vetbact website, 2020); *Mycoplasma* which is present in the oral cavity and urogenital tract (vetbact website, 2020); *Oligella* which is a commensal of the urogenital tract and may cause opportunistic infections (Roussau et al., 1987); *Kocuria* which are isolated from the human oropharynx (Al‐Kass et al., 2018); and *Psychrobacter*, which is found in water but has also been isolated occasionally from human beings (vetbact website, 2020). A potential pathogen, *Pseudomonas* spp., was isolated in low numbers (1 or 2 colony forming units /mL) in three samples (Blanchard et al., 1992).

Similarly, in semen from stallions in Group B, only Staphylococcus appeared in all six animals tested. Other bacteria appeared in some semen samples but not in others e.g. *Bacillus, Corynebacterium* and *Micrococcus*. The remaining bacteria were isolated in semen from only one of the six stallions, e.g. with semen from stallion 4 having three of these taxa. Stallions 2, 5 and 6 had only one taxa each (*Fusobacterium, Pantoea* and *Mycoplasma*, respectively). *Fusobacterium* is isolated from the human oropharynx (vetbact, website, 2020).

Fewer taxa were isolated from the semen samples from stallions in Group B than in Group A. Interestingly, the six samples in Group B were taken at the start of the breeding season, whereas the samples from Group A were taken at the end of the breeding season. These findings are in agreement with a previous study in Germany (Pasing et al., 2013), in which the number of microbial species in semen and the intensity of growth increased from April to August, i.e. during the breeding season. More taxa were isolated in our study during July/August than in the study by Pasing & Aurich, although only blood agar, nutrient agar and Gassner agar were used to culture the bacteria in their study. Therefore, more organisms might have been present than were cultured. Straw or wood chippings were used as bedding for their stallions, whereas shavings (Group A) or straw (Group B) were used for the stallions in the present study.

These observations could imply that the bacterial flora in the genital tract may depend more on the individual than on the environment in which the stallion is kept. However, a confounding factor is that the microbes present in a community are modulated by the microbes themselves (Braga, Dourado & Araujo, 2016); coexistence is a result of interactions between microbes as well as between the host and the microbes (Niehaus et al., 2019). The host/microbe interaction may alter host physiology (Braga et al., 2016), which in the case of the genital tract, could have an effect on sperm quality. However, no effect of the presence of particular microbes on sperm quality was detected in the study by (Pasing et al., 2013).

A number of bacteria could not be identified as their spectra were not contained in the MALDI-TOF database. The types of bacteria identified varied between these different studies, possibly because different methods were used for their isolation and identification, but also because of different husbandry and environmental conditions. Most other studies did not report the husbandry conditions of the stallions. A study in Brazil reported that microbial load was reduced when penile washing was not carried out prior to semen collection (Neto et al., 2015); however, other husbandry conditions were not reported in their study. The occurrence of bacteria in the extender has been observed previously (Al-Kass et al., 2019; Morrell, Klein, Lundeheim, Erol & Troedsson, 2014). Therefore, low numbers of these bacteria in the extended semen samples may have originated from the extender rather than from the semen itself e.g. *Bacillus* spp., and Micrococcus in one stallion, or they could have been contaminants from the personnel during preparation of the semen samples, or from the environment in the laboratory at the stud. Although the semen collector wore gloves, it is not known if the person preparing the semen extender and extending the semen also wore gloves. The results should be interpreted with care where only low numbers of bacteria are present in both extender and extended semen. However, typing of the isolates by whole genome sequencing would be required in order to determine if the extender was the source of these particular bacteria, which was beyond the scope of the present study. The other microorganisms isolated from the extender, *Micrococcus, Corynebacterium* spp., *Mycoplasma* spp., and *Staphylococcus* spp., were mostly present in much higher numbers in the extended semen than in the extender itself, suggesting that they were present in semen prior to the addition of the extender.

The number of different agar plates and incubation conditions used in the present study are more than usually used for general bacteriology of semen samples, as described by others [e.g. Al-Kass et al., 2019]. In the laboratory at SVA, for example, routine microbiology on semen would be done with horse blood agar, bromocresole lactose purpur agar and *Pseudomonas* isolation agar plates (Al-Kass et al., 2019). It would be impractical and too expensive to follow the protocol used in the present study on a routine basis, but it was interesting to see how many additional taxa could be isolated using a wide variety of plates and culture conditions. One example is that *Corynebacterium* were isolated in large numbers from stallion 2 using FAA whereas none were isolated on blood agar. Even so, it was not possible to identify 36% of the isolated bacteria because the remainder were not found in the MALDI-TOF database. Updating the software may facilitate identification of more bacteria. In addition, non-viable bacteria cannot be identified with this method, since they must first be cultured to obtain material for MALDI-TOF MS. In order to detect these bacteria as well, researchers are looking for alternative methods such as Flow Cytometry (Gunasekera, Attfield & Veal, 2000; Wu, Wang, Song, Wang & Yan, 2016) or 16S sequencing of extracted DNA which can identify very low bacterial loads, and non-viable or damaged bacteria as well as live bacteria (Ben-Amor et al., 2005; Kuczynski et al., 2012; Xu, 2014). The 16S sequencing method identifies all DNA associated with a particular sequence unique to bacteria, and therefore can be used to identify DNA even from non-viable bacteria. From the point of view of stored semen samples, it is important to know if non-viable as well as viable bacteria are present, since they can have a negative effect on sperm quality.

## Conclusions

*Staphylococcus* spp. were isolated from all samples. Potential pathogens were absent, with the possible exception of *Pseudomonas* spp. present in very low numbers in three of the extended semen samples. Half of the bacterial taxa isolated originate from skin and mucus membranes. Many environmental bacteria were isolated, but the taxa varied among animals from the same stud as well as among animals on different studs. Some bacteria appeared to have originated from the extender itself or during its preparation. Microbes usually found in the human oropharynx were isolated in some semen samples; it is not known whether they are also present in the equine oropharynx. It may be advisable for personnel to wear masks as well as gloves when collecting and handling semen. Knowledge of the bacterial taxa present in stallion semen is essential for developing protocols for best praxis in semen handling. A further investigation into the factors potentially affecting the microbial population, such as season, or husbandry conditions, would facilitate development of such protocols. Many isolates were cultured on the different specialist agars but it was not possible to identify all of them by MALDI-TOF MS; therefore, future studies may need to utilize more advanced methods of microbial identification.

## Declarations

Ethics: not ethical approval is required in Sweden for collection of stallion semen using an artificial vagina at the present time.

Consent for publication: all authors have agreed to the publication of this manuscript

Data availability: All data from this study are presented in the manuscript.

## Funding

This study was sponsored by the ministry of Higher Education and Scientific Research, Iraq (Z.A.-K.) and the veterinary faculty at the Swedish University of Agricultural Sciences.The sponsors did not play a role in study design; in the collection, analysis and interpretation of data; in the writing of the report; or in the decision to submit the article for publication.

## Ethical statement

The study did not involve an experiment using animals. We obtained semen from a stud where stallions are kept for semen collection by artificial vagina, which does not require ethical approval in Sweden at the present time. The animals are housed and cared for according to national and international regulations.

## Declaration of Competing Interest

The authors do not have any conflict of interest to declare.
